# Diabetes Mellitus: Its Pathological Chemistry and Treatment

**Published:** 1906-09

**Authors:** 


### Reviews of Books.

**Diabetes Mellitus: its Pathological Chemistry and Treatment.** By CARL VON NOORDEN. English edition by FLORENCE BUCHANAN, D.Sc., and I. WALKER HALL, M.D. Pp. 201. Bristol: John Wright & Co. 1906.

If anyone wants to know of the recent work done upon this subject, and of the applications of the advances in physiological and pathological chemistry to the disease, let him go out straightway and purchase this book. True, he must swallow the pill in the dogmatic covering von Noorden always provides, but the acidity of the reviewers will soon dissolve the outer coat. To some of the reviews already published the reader may turn for such critical consideration, and also for a digest of the book, which a few may prefer even to the book itself. Here it must suffice to state that the text includes a discussion upon the pathogenesis of glycosuria, non-diabetic as well as diabetic, of the acetone bodies, of the energy balance and the nitrogenous balance, of the general course and prognosis, of the treatment of diabetes, and an appendix of permissible foods and their analyses.

This volume is quite another type of book from that of the other numbers of this series. It consists of the lectures delivered in the University and Bellevue Hospital Medical College, New York, in November, 1905. Thanks to the translators, the English is eminently readable, and quite suitable for armchair perusal, since there is an admirable lack of Teutonic sentences. There is really much that is suggestive in the way of treatment. Some reader, perhaps, like the reviewer, may sigh as he reaches the last page for more opportunities for making practical use of the hints given.

